# Buckling versus Crystal Expulsion Controlled by Deformation Rate of Particle-Coated Air Bubbles in Oil

**DOI:** 10.1021/acs.langmuir.1c03171

**Published:** 2022-01-13

**Authors:** Saikat Saha, Francis Pagaud, Bernard P. Binks, Valeria Garbin

**Affiliations:** † Department of Chemical Engineering, Delft University of Technology, 2629 HZ Delft, The Netherlands; ‡ Department of Chemical Engineering, Imperial College London, London SW7 2AZ, United Kingdom; § Department of Chemistry, University of Hull, Hull HU6 7RX, United Kingdom

## Abstract

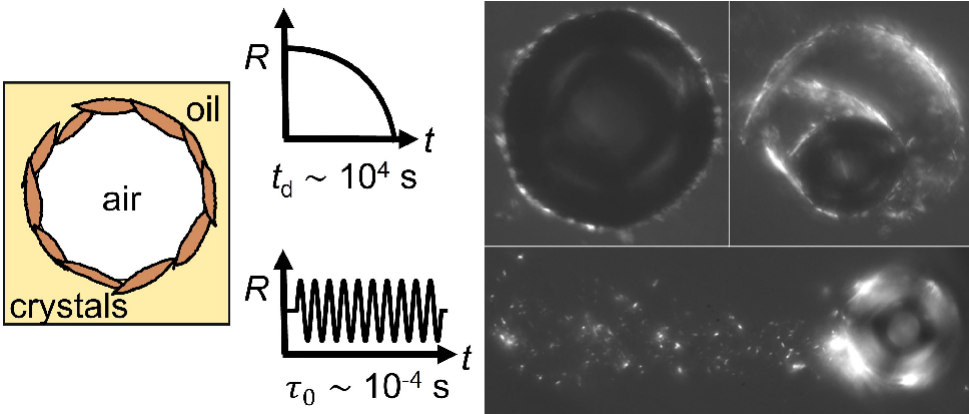

Oil foams stabilized by crystallizing agents exhibit outstanding stability and show promise for applications in consumer products. The stability and mechanics imparted by the interfacial layer of crystals underpin product shelf life, as well as optimal processing conditions and performance in applications. Shelf life is affected by the stability against bubble dissolution over a long time scale, which leads to slow compression of the interfacial layer. In processing flow conditions, the imposed deformation is characterized by much shorter time scales. In practical situations, the crystal layer is therefore subjected to deformation on extremely different time scales. Despite its importance, our understanding of the behavior of such interfacial layers at different time scales remains limited. To address this gap, here we investigate the dynamics of single, crystal-coated bubbles isolated from an oleofoam, at two extreme time scales: the diffusion-limited time scale characteristic of bubble dissolution, ∼10^4^ s, and a fast time scale characteristic of processing flow conditions, ∼10^–3^ s. In our experiments, slow deformation is obtained by bubble dissolution, and fast deformation in controlled conditions with real-time imaging is obtained using ultrasound-induced bubble oscillations. The experiments reveal that the fate of the interfacial layer is dramatically affected by the dynamics of deformation: after complete bubble dissolution, a continuous solid layer remains; after fast, oscillatory deformation of the layer, small crystals are expelled from the layer. This observation shows promise toward developing stimuli-responsive systems, with sensitivity to deformation rate, in addition to the already known thermoresponsiveness and photoresponsiveness of oleofoams.

## Introduction

Oil foams have diverse use in pharmaceutical, food, and cosmetic applications.^1,2^ While air–oil interfaces are difficult to stabilize with molecular surfactants,^3^ various crystallizing agents have been found to confer long-term stability to oil foams^2−8^ and novel air-in-oil-in-water systems.^9^ Crystals can nucleate and grow both in the bulk oil phase and directly on the oil–air interface, upon decreasing the temperature of a preheated solution.^10^ Crystals formed in the bulk can then adsorb to the air–oil interfaces during mixing, while excess crystals can form a network in the oil phase (*i.e.*, an oleogel).^5^ In this case, foam stability is due to both the bulk and interfacial networks of crystals.^11,12^ Much progress has been made in understanding the formation and stability of oil foams stabilized by crystallizing agents, yet our knowledge of the mechanical properties and dynamic behaviors of such systems remains limited, despite its importance for product shelf life, as well as optimal processing conditions and performance in applications. In particular, while there is significant literature available on the rheology of oleogels formed by the crystal network in the bulk oil phase,^1,2^ and on the link between crystal formation and network properties,^13,14^ less is understood about the microstructure and properties of the crystal-stabilized interfaces.^15^

The stability, dynamics, and mechanics of particle-stabilized (or “armored”) bubbles^16^ have been studied extensively for model systems stabilized by micrometer-scale colloids; these studies provided important fundamental insights thanks to the ability to directly visualize the interface microstructure and its evolution. At high surface coverage, particle jamming at the interface supports anisotropic stresses, which oppose the shape-minimizing effects of surface tension and lead to stable, non-spherical bubbles.^16^ If the particles have attractive interactions, the sufficient condition to arrest bubble dissolution depends on the surface viscoelasticity^17^ rather than surface coverage. Such attractive interactions can be tuned by modifying the particle shape, size, roughness, or surface chemistry.^17^ In addition to surface coverage and interparticle interactions, the morphology and deformation of the interfacial monolayer can also depend on the particle-to-bubble size ratio.^18−21^ Upon forced dissolution of particle-stabilized bubbles, in both aqueous^22^ and nonaqueous^23^ media, either expulsion of particles or detachment of a continuous layer from the interface was observed, while for ultrafast deformation of colloid-coated bubbles in water forced by ultrasound, buckling of the monolayer and particle expulsion were observed.^24^

In this work, we explore the dynamic response to deformation of wax crystal stabilized bubbles isolated from the bulk crystal network of an oleofoam. We exploit bubble dissolution to apply slow interfacial compression on a diffusion-limited time scale, 10^3^–10^4^ s, important for product stability and shelf life.^25^ Using ultrasound-driven bubble oscillations, we apply compression/expansion on a very fast time scale, 10^–4^ s, relevant in production processes^26^ and performance in applications. The experiments reveal a striking difference in the fate of the interfacial wax crystal layer at these two extreme deformation time scales. These insights are also important for optimizing strategies for the design, synthesis, and application of stimuli-responsive systems stabilized by phase-changing materials.^2,27^

## Materials and Methods

### Preparation of Wax Crystal-Coated Bubbles

The wax used (Hydropel QB, Shamrock Technologies) is a blend of paraffin and synthetic waxes with a melting temperature range *T*_m_ = 50–105 °C, according to the manufacturer’s specifications. The wax particles were used as received. Consumer-grade sunflower oil (Tesco) was used as received. The density was measured to be ρ_oil_ = 0.8879 g cm^–3^ and the viscosity η_oil_ = 50 mPa s at *T* = 25 °C. The protocol for oleofoam preparation was provided previously.^11^ Briefly, a vial containing 2.5 wt/vol % wax particles in sunflower oil was agitated at 3000 rpm for 2 min to form a well-mixed suspension. The suspension was then heated to *T* = 90 °C to melt the wax and immediately agitated for 2 min to incorporate air to form the oleofoam. During turbulent mixing, the sample cooled to *T* ≈ 45 °C. The vial was then left undisturbed to cool to room temperature. In this process, the excess wax crystals that are not incorporated on the interfaces of bubbles form a gel network in the oil phase (oleogel) which contributes to the stability of such systems.^11^

**Figure 1:**
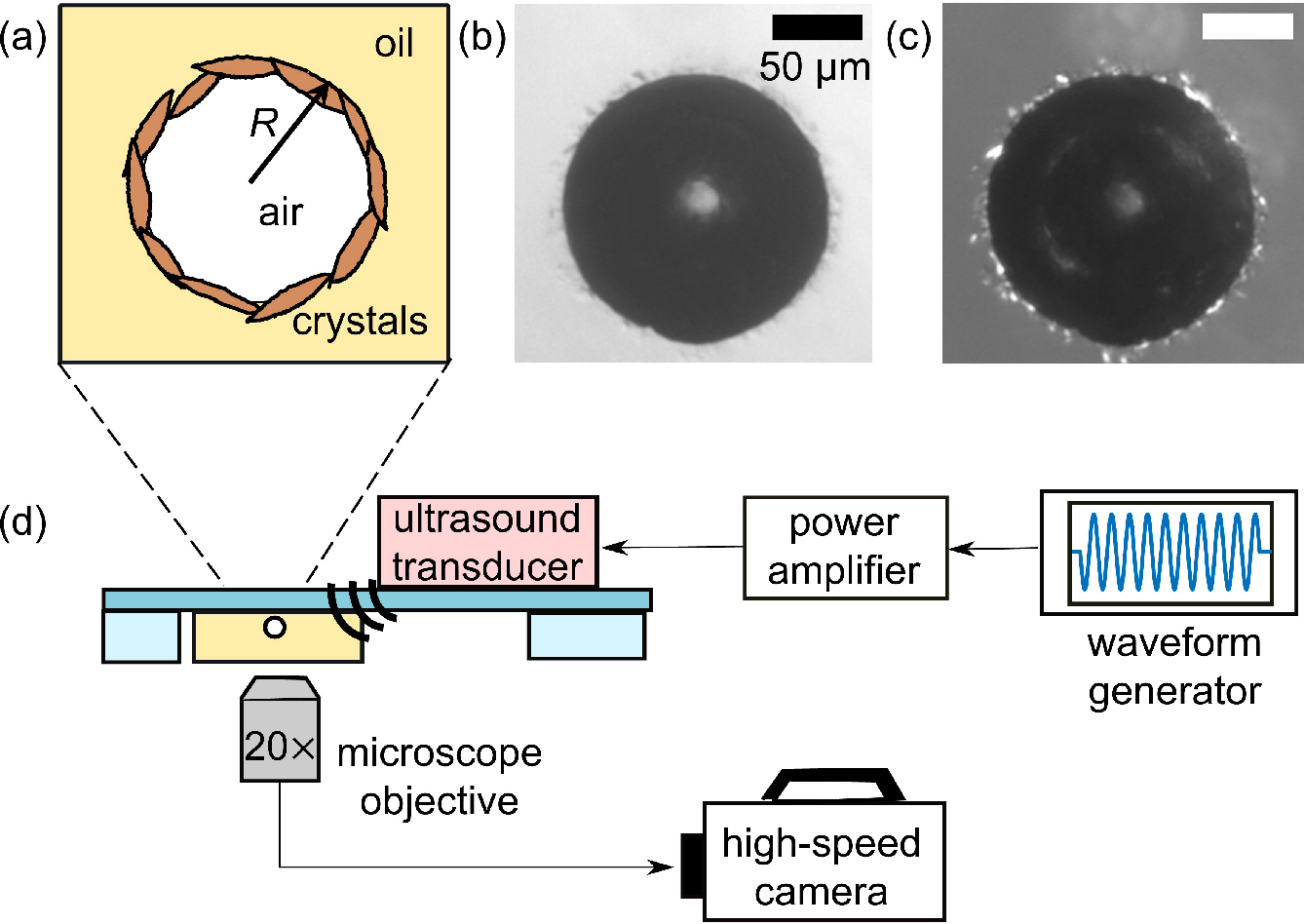
(a) Schematic of an air bubble stabilized by wax crystals in oil. (b) Wax-coated bubble viewed using bright-field microscopy. (c) The same bubble viewed under crossed polarizers. (d) Schematic of the experimental setup.

To prepare samples with wax-coated bubbles without the bulk gel network, the prepared oleofoam was diluted by placing a small sample (≈20 μL) on a microscope glass slide with a spacer of thickness 1.5 mm and adding sunflower oil to obtain isolated bubbles as schematically shown in Figure 1a. High-magnification optical micrographs suggest that the primary crystal size at the oil/air interface is in the range 1–10 μm (see the Supporting Information).

The wax-coated bubbles, when extracted from the oleofoam and re-suspended in sunflower oil, still have their interfacial layers present, as confirmed by their textured interfaces and buckled shapes, visible in Figure 1b, recorded in bright field with an inverted microscope (IX71, Olympus) and digital camera (DCC1645C, Thorlabs). Using crossed polarizers, the layer of crystals is more clearly visible, as shown in Figure 1c. Wherever these crystals can be seen, the interface exhibits non-uniform curvature. Conversely, in regions devoid of crystals, the curvature is uniform. This is characteristic of a cohesive interfacial layer, which does not expand to fill the entire available area.

### Bubble Dissolution Experiments

We exploit the phenomenon of bubble dissolution to access long time scales of interface deformation. In the absence of an interfacial layer, the characteristic time scale for bubble dissolution can be estimated^28^ by integrating the equation governing the rate of change of radius from the Epstein and Plesset theory^29^ to give
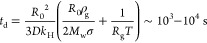
1

This long time scale is achieved by selecting bubbles with an initial radius *R*_0_ ≈ 100 μm. For such small bubbles, the Laplace pressure of the gas–liquid interface, 2σ/*R*_0_ with σ ≈ 30 mN/m for air–oil interfaces,^25^ accelerates dissolution; this effect is described by the first term between parentheses in eq 1. The physicochemical properties affecting *t*_d_ are the gas diffusivity in the oil, *D* ∼ 10^–10^ m^2^/s for vegetable oils,^25^ and the solubility of the gas in the oil, measured by Henry’s constant *k*_H_, which for N_2_ in nonpolar solvents^23^ is on the order of *k*_H_ ∼ 10^–5^ mol Pa^–1^ m^–3^. The other parameters are the gas density and molar mass, which for nitrogen are ρ_g_ = 1.2 kg/m^3^ and *M*_w_ = 28 × 10^–3^ kg/mol, respectively; the temperature *T* = 298 K; and the universal gas constant *R*_g_ = 8.31 J mol^–1^ K^–1^.

For wax-coated bubbles, after they are extracted from the bulk oleogel, the interfacial layer alone is not able to arrest bubble dissolution.^11^ The stability imparted by the interfacial layer depends on the initial surface coverage and microstructure, and a large variability in dissolution time *t*_d_ of these crystal-coated bubbles is observed. Typically, dissolution occurs over a time scale in the same range as for uncoated bubbles, *t*_d_ ∼ 10^3^–10^4^ s.

### Oscillation of Bubbles Using Ultrasound

To access short time scales of interface deformation, we exploit ultrasound-driven bubble oscillations. The periodic oscillations of the pressure around the ambient value, *p*_0_, caused by an acoustic wave, force the gas core of a bubble to undergo periodic compression and expansion at the same frequency.^30^ The driving frequency *f* was selected close to the natural frequency *f*_0_ of a bubble of radius *R*_0_ in an unbounded liquid. The relation between these quantities is given by the Minnaert frequency,^30^ modified to include surface tension effects:
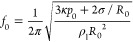
2where κ is the polytropic exponent of the gas, taken to be 1.4 (assuming adiabatic behavior); σ is the surface tension; and ρ_l_ is the liquid density. For bubbles of radii *R*_0_ ≈ 100 μm, the driving frequencies were selected in the range *f*_0_ ∼ 10 kHz, leading to oscillatory deformation with a period
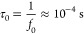
3

To transmit ultrasound waves into the sample and observe the dynamics of wax-coated bubbles, we used an ultrasound transducer (bolt-clamped Langevin transducer, Steminc; resonance frequency 25 kHz) glued onto a glass plate and driven by an arbitrary waveform generator (Agilent 33220A, Agilent Technologies, Inc.) connected to a linear power amplifier (AG 1021, T&C Power Conversion), as shown in Figure 1d. A sine wave of prescribed frequency *f*, acoustic pressure amplitude Δ*p*, and number of cycles *N* was sent to the transducer by the waveform generator and amplifier. The ultrasound-driven bubble dynamics are recorded with a high-speed camera (FASTCAM SA5, Photron) connected to the inverted microscope. The frame rate used for the high-speed camera was at least 10 times the driving frequency. The amplitude of bubble oscillations, which is controlled both by the forcing frequency *f* and by the acoustic pressure amplitude Δ*p*, is quantified from image analysis as *x*_0_ = Δ*R*/*R*_0_, with Δ*R* the maximum amplitude of bubble oscillations and *R*_0_ the equilibrium bubble radius.

## Results and Discussion

### Experimental Observations

**Figure 2:**
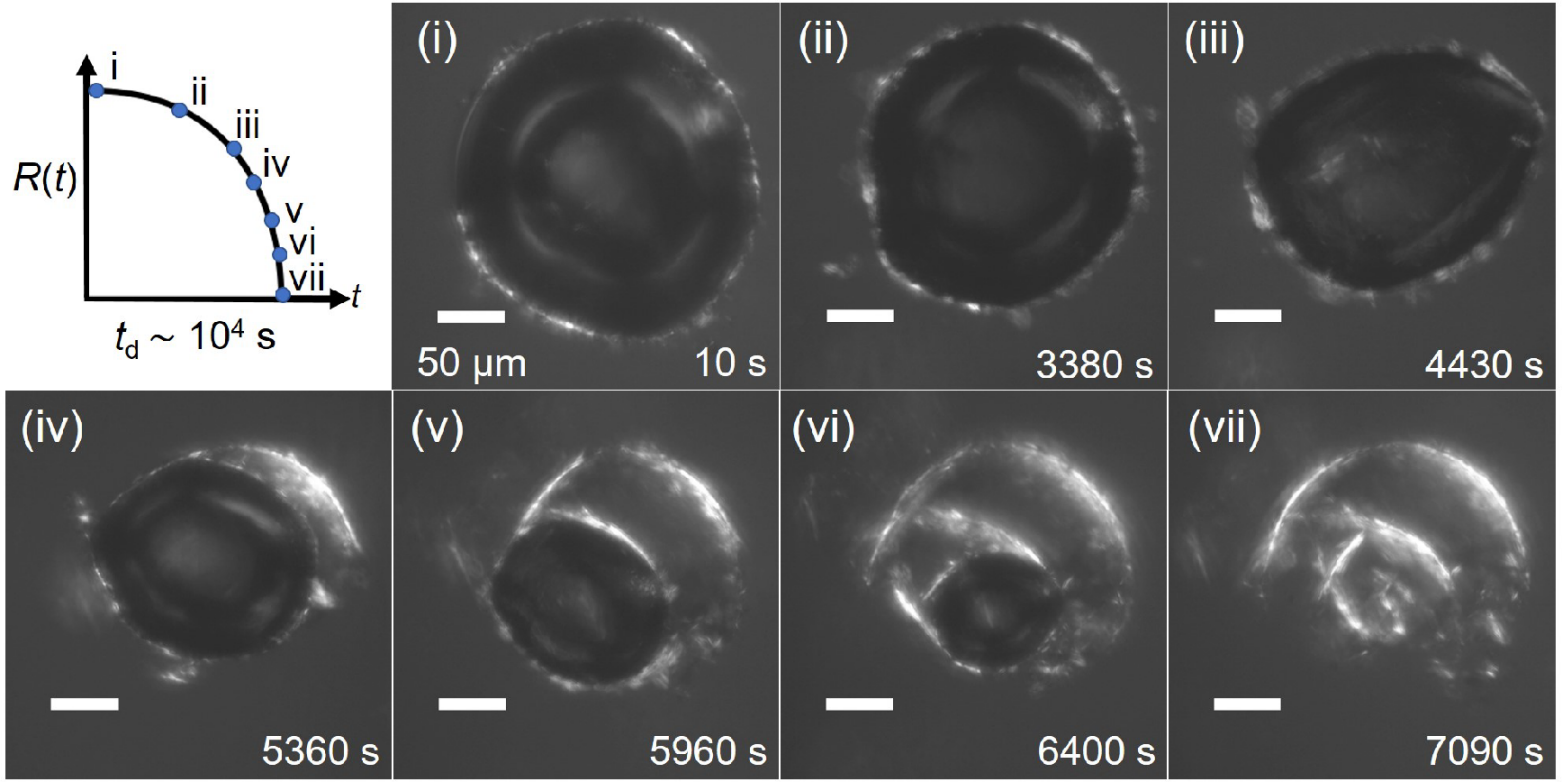
Dissolution of wax crystal-coated bubble in oil (SI Video 1). Schematic of bubble dissolution, occurring over *t* ∼ 10^3^–10^4^ s, with points qualitatively marking the dissolution steps observed in the following panels (i–vii). The image sequence is taken using crossed polarizers.

The dynamics of dissolution of a bubble in oil, stabilized by an interfacial layer of wax crystals, is shown in Figure 2 (Supporting Information (SI) Video 1). The schematic shows qualitatively the time evolution of the radius *R*(*t*), and the different stages of the process are shown in images i–vii, which were taken with crossed polarizers to highlight the layer of crystals (seen as bright in the images). Between *t* = 4430 s and *t* = 5360 s (Figure 2iii,iv), a sharp deformation of the interface is observed on the right side of the bubble, from where a cohesive layer of crystals detaches as the bubble dissolves. Between *t* = 5960 s and *t* = 6400 s (Figure 2v,vi), there is again a sharp distortion to the left side of the bubble, followed by detachment of the layer from that location. Thus, detachment appears to initiate from the points of maximum deformation. At *t* = 7090 s, after the bubble has completely dissolved (Figure 2vii), a cohesive layer of crystals, seen as a bright spiral-like structure in the image, is left behind. The layer of crystals was observed to remain intact over months. The layer appears to be composed of large crystal rafts with characteristic dimension *l* comparable to the initial bubble radius (*R*_0_ ≈ 100 μm).

**Figure 3:**
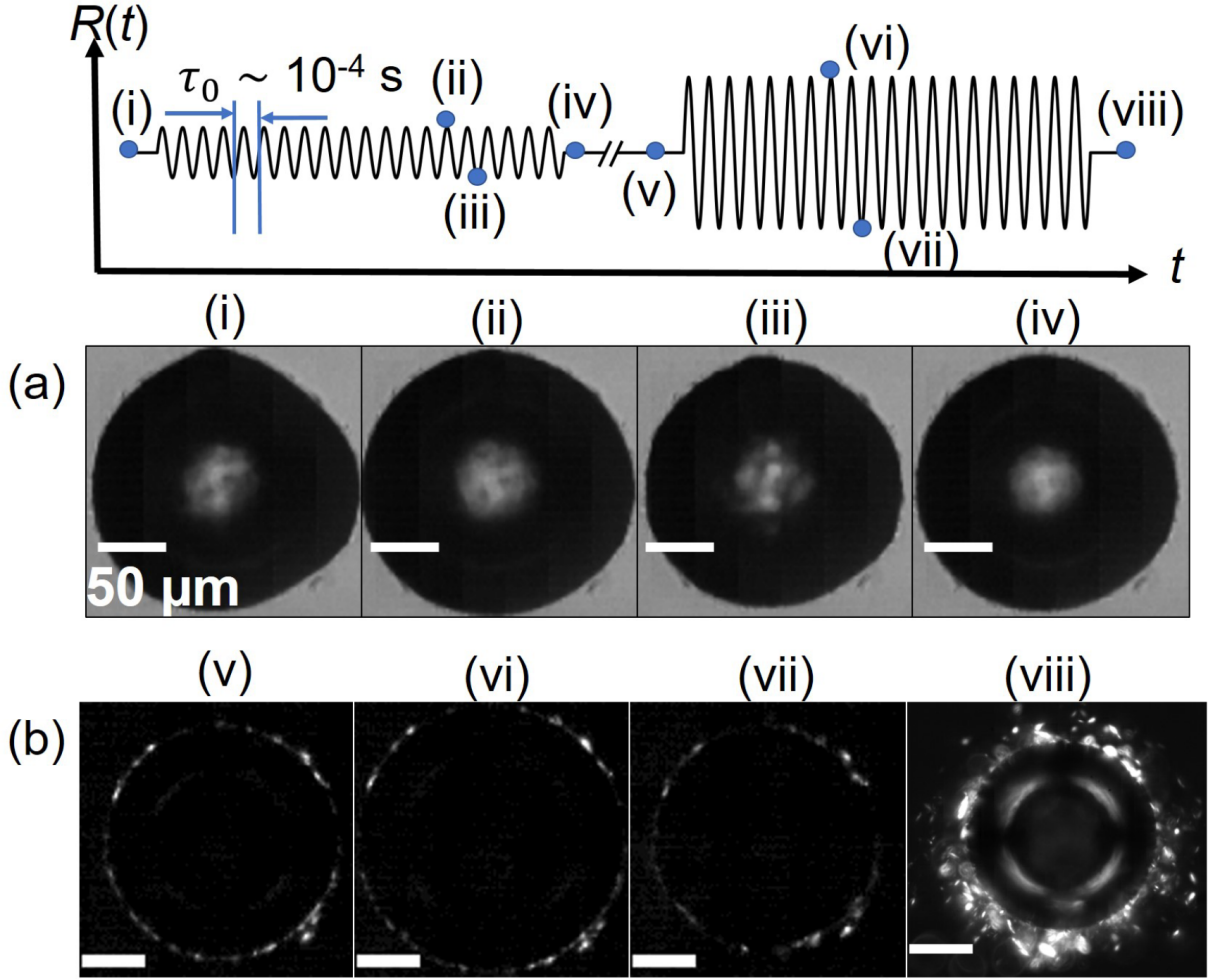
Crystal expulsion after ultrasound-driven bubble oscillations. The schematic illustrates the protocol of oscillating a crystal-coated bubble with ultrasound pulses of different amplitudes. Images at selected times, labeled (i)–(viii), are shown below. (a) High-speed image sequence of a wax-coated bubble with initial radius *R*_0_ = 101 μm undergoing oscillations of moderate amplitude, with *x*_0_ = Δ*R*/*R*_0_ ≈ 3%, *N* = 50 cycles (SI Video 2). The frames show (i) the initial non-spherical shape of the bubble before oscillations, the (ii) maximum and (iii) minimum during oscillations at *f* = 25 kHz, and (iv) the final, almost spherical shape after a total of *N* = 260 cycles of oscillations of small amplitude. (b) High-speed image sequence with crossed polarizers, for large-amplitude oscillations, with *x*_0_ ≈ 20% (SI Video 3): (v) initial state before oscillations, (vi) maximum and (vii) minimum during oscillations at *f* = 25 kHz, and (viii) final state after *N* = 100 cycles of oscillations, showing expulsion of crystals around the bubble.

**Figure 4:**
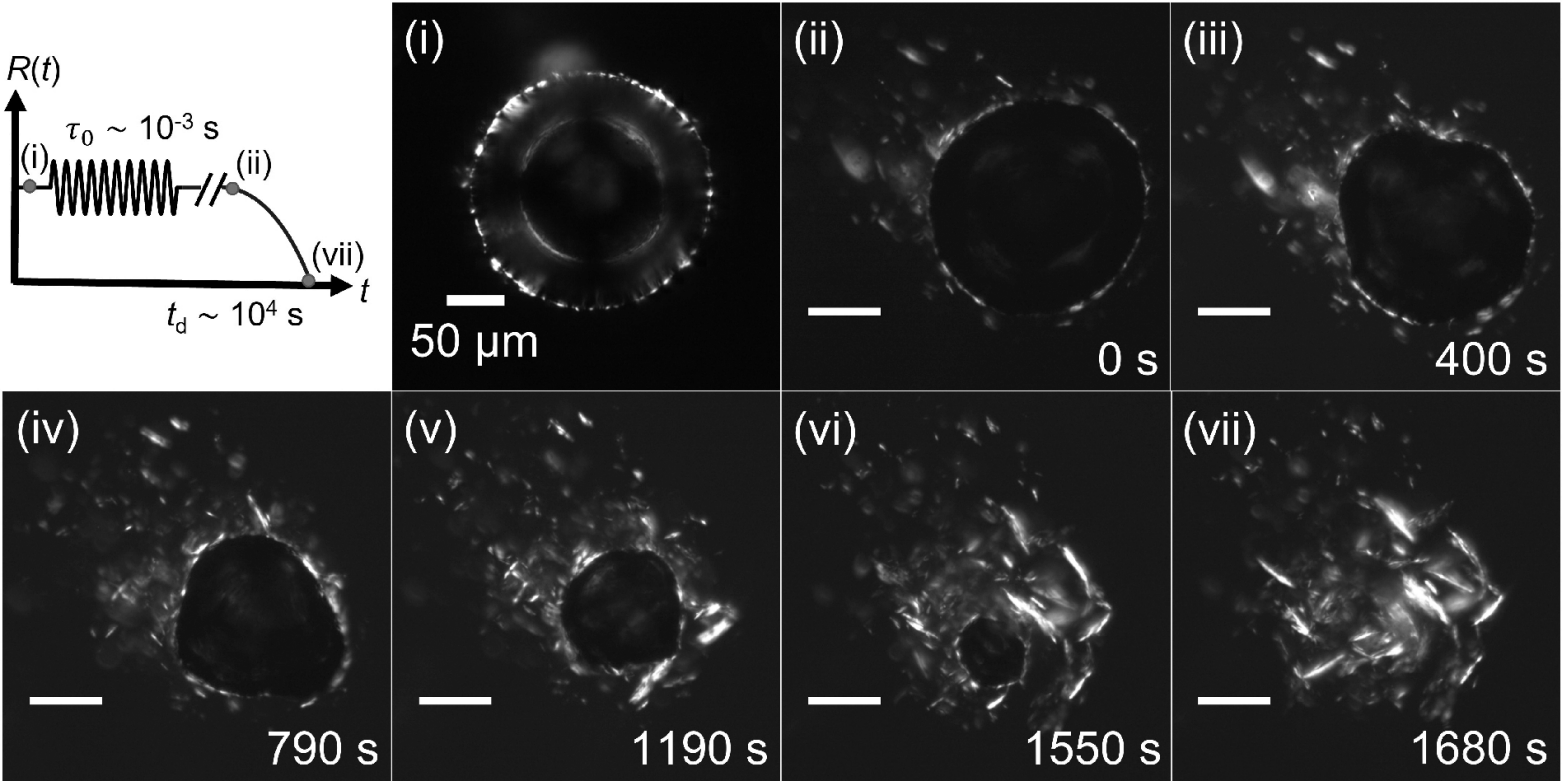
Bubble dissolution after ultrasound-driven bubble oscillations. The schematic shows the protocol including large-amplitude radial oscillations, followed by unperturbed dissolution. The labels correspond to the image sequence shown in panels i–vii. The initial radius of the bubble before oscillations (i) is *R*_0_ = 102 μm. Oscillations are driven by ultrasound at *f* = 25 kHz for *N* = 480 cycles, with amplitude of oscillations *x*_0_ > 20% (shown in SI Video 4). (ii) *t* = 0 s represents the start time of recording as the bubble dissolves (ii–vii).

Figure 3 shows the effects of ultrasound-driven bubble oscillations of small and large amplitudes on the same wax-coated bubble. The frequency of oscillations is *f* = 25 kHz, and the bubble is successively excited by pulses of *N* cycles (two pulses of different amplitudes are shown in the schematic of Figure 3 by way of example). At moderate acoustic pressure, the amplitude of bubble oscillations is limited to *x*_0_ ≈ 3%, too small to be clearly visible in Figure 3a, and more clearly visible in the corresponding SI Video 2, which shows a pulse of *N* = 50 cycles. The interface buckles during compression (Figure 3a-iii) and, over a total of *N* = 260 cycles, undergoes a clear change from the initial non-spherical shape (Figure 3a-i) to almost spherical shape (Figure 3a-iv). For the same bubble at higher acoustic pressures, the amplitude of oscillations becomes *x*_0_ > 20%, which is clearly visible in the high-speed recording with crossed polarizers (SI Video 3) as well as in the corresponding image sequence in Figure 3b. The morphology of the crystal layer after large-amplitude oscillations at high frequency (*N* = 100 cycles) is profoundly different from the case of slow compression during bubble dissolution: crystals are expelled from the layer, which are much smaller than the initial bubble radius, *l* ≪ *R*_0_, seen in Figure 3b-viii. In contrast, the size of the segments forming the cohesive layer after bubble dissolution is *l* ≈ *R*_0_. Lastly, we examined the effect of ultrasound-driven oscillations on subsequent dissolution of wax-coated bubbles. Following a period of ultrasound-driven oscillations, the bubbles were left to dissolve completely, as shown in the schematic in Figure 4. Figure 4i shows a wax-coated bubble of initial radius *R*_0_ ≈ 102 μm before any deformation is applied. The bubble was first driven into large-amplitude oscillations (*x*_0_ > 20%) with ultrasound frequency *f* = 25 kHz, and for *N* = 480 cycles. After this number of cycles of oscillations, expulsion of a limited amount of crystals from the interface was obtained, without complete disruption of the interfacial layer, as seen in Figure 4ii. Forcing with ultrasound was stopped at this point, and the same bubble was then left to dissolve. An image sequence of the bubble dissolution steps is shown in Figure 4ii–vii (see also SI Video 3). The crystals and fragments of interfacial layer left behind after the bubble has completely dissolved (*t* = 1680 s; see Figure 4vii) appear larger compared to those that are expelled by ultrasound-driven oscillations; see Figure 3viii for comparison.

### Mechanism of Layer Detachment for Slow Compression

Observations of the dissolution of wax-coated bubbles (sample size *n* ≈ 10) indicate that, as the bubble decreases in size, the interfacial layer gradually detaches from the air–oil interface without significant changes in its morphology. In some instances (*n* = 3), the bubble remains fully covered with an interfacial layer during the entire process, until its complete dissolution (see Figure 2). These observations suggest that the energy penalty of deformation of the interfacial layer, measured by the compression modulus, exceeds the adhesive interaction energy between the wax layer and the air–oil interface. The compression modulus of the wax crystal layer on an air/oil interface was previously estimated^11^ to be *K*_d_ ≈ 40–60 mJ/m^2^. As the interfacial layer detaches from the bubble interface, the inner surface of the (solid) wax crystal layer, initially in contact with air, becomes wetted by oil and a region of bare oil/air interface is formed. In this process, the change in interfacial energy per unit area can therefore be estimated to be in the range γ_os_ + γ_oa_ – γ_as_ ≈ 1–10 mJ/m^2^, with γ_oa_ ≈ 30 mJ/m^2^ the surface tension of the oil/air interface, γ_os_ ≈ 1–5 mJ/m^2^ the interfacial energy of the solid/oil interface, and γ_as_ ≈ 20–35 mJ/m^2^ the surface energy of the solid/air interface. It is therefore energetically favorable for the layer to detach from the gas interface during bubble dissolution, since the change in interfacial energy is small compared to the compression modulus. Comparing with the literature for clay-stabilized bubbles in alkanes^23^ and latex-stabilized bubbles in water,^22^ we identify two features common to all systems for when a continuous layer is left behind: the interface deformation is slow, occurring over a time scale *t*_d_ ∼ 10^3^–10^4^ s, and the size of the particles is much smaller than the bubble radius, leading to a continuum behavior.^21^

### Mechanisms of Crystal Expulsion for Fast Deformation

During ultrasound-driven bubble oscillations, the wax crystal layer undergoes rapid compression and expansion, as well as out-of-plane deformation due to buckling, shown in Figure 3a-iii, which can break the initially cohesive layer. This hypothesis is supported by the observation in Figure 3a that, for small-amplitude oscillations, the shape of the bubble changes without expulsion of interfacial material. The bubble becomes less non-spherical, consistent with an interfacial microstructure that cannot support anisotropic stresses. Because compression occurs on a very short time scale, τ_0_ ∼ 10^–4^ s, compared to rearrangement time scales in densely packed colloid monolayers, there is not sufficient time for rearrangement of the crystals to form a cohesive network. When a bubble that has been pre-treated with ultrasound-driven oscillations is allowed to dissolve (Figure 4), the interface undergoes slow compression, allowing sufficient time for the crystals to rearrange and form again a cohesive, solid-like network that can support anisotropic stresses as evidenced by the non-spherical shape of the bubble (Figure 4iii–v).

For bubble oscillations of large amplitude, several concurrent mechanisms can contribute to the expulsion of crystals, similar to observations for colloid-coated bubbles in water,^24^ including significant out-of-plane buckling of the layer (SI Video 5). In the case of large-amplitude oscillations, when the same bubble is allowed to dissolve, the fragments of interfacial layer left behind are larger than those produced by ultrasound oscillations alone. This suggests that the large-amplitude oscillations can break the original interfacial structure but, following this, as the interface is compressed slowly there is sufficient time for the remaining crystals to rearrange and form larger cohesive rafts.

Finally, a secondary, slow flow called “microstreaming” and caused by bubble oscillations in confined geometries^31^ cannot be ruled out in our experiments, but is not expected to disrupt the interfacial layer. We do not see direct evidence of microstreaming flow, but its effect would be to re-distribute expelled crystals along characteristic, closed streamlines around the bubble.

## Conclusions

We investigated the response to deformation of an interfacial layer formed by a crystallizing agent on the surface of bubbles in an oil phase, at two extreme time scales that are, on one hand, relevant to the shelf life of such systems and, on the other hand, relevant to their processing and application. We found that the morphology of the interfacial layer is modified, depending on the interplay between the time scales of deformation and crystal rearrangement. For slow deformation, the crystals form a cohesive network, whereas for fast deformation the layer is fragmented. Conversely, the fate of the monolayer can be controlled by carefully combining these deformation protocols on the same layer. Also, depending on the requirement, the bubble shapes can be altered. These effects can find utility in modifying the morphology, rheology, and texture of foams in cosmetic and food applications. Furthermore, by tuning the timing and duration of ultrasound-induced deformation, protocols can be devised for the use of crystal-coated microbubbles for controlled release, useful for delivery of active pharmaceutical ingredients. Our findings pave the way for the design of stimuli-responsive systems that can be triggered to behave uniquely depending on the time scale of deformation, in addition to the already-known temperature dependence of the phase-changing stabilizing agent.

## Supporting Information Available

High-magnification optical micrographs and characterization of crystal shape and size in bulk oleogel network and at oil/air interface (PDF)
Dissolution of a wax-coated bubble in oil, imaged with crossed polarizers (MP4)
Small-amplitude oscillations (driven by ultrasound at 25 kHz) of a wax-coated bubble in oil (MP4)
Large-amplitude oscillations (driven by ultrasound at 25 kHz) of a wax-coated bubble in oil, imaged with crossed polarizers (AVI)
Dissolution of a wax-coated bubble after pre-treatment with large-amplitude ultrasound-driven oscillations, imaged with crossed polarizers (MP4)
Large-amplitude oscillations (driven by ultrasound at 25 kHz) of a wax-coated bubble showing buckling (AVI)

## Supplementary Material

la1c03171_si_003.mp4

la1c03171_si_004.avi

la1c03171_si_005.mp4

la1c03171_si_006.avi
