# Supplementary material for: Buckling versus Crystal Expulsion Controlled by Deformation Rate of Particle-Coated Air Bubbles in Oil
Source: Langmuir. 2022 Jan 13;38(3):1259–65. doi: 10.1021/acs.langmuir.1c03171 (PMC8793140; doi:10.1021/acs.langmuir.1c03171)
Supplement: Supplementary file 1 — la1c03171_si_001.pdf [file la1c03171_si_001.pdf]

Supporting information

Buckling *versus* crystal expulsion controlled by deformation rate of particle-coated air bubbles in oil

Saikat Saha,[†,‡] Francis Pagaud,[‡,§] Bernard P. Binks,[¶] and Valeria Garbin*[,†,‡]

†*Department of Chemical Engineering, Delft University of Technology, 2629 HZ Delft, The Netherlands.*

‡*Department of Chemical Engineering, Imperial College London, London SW7 2AZ, United Kingdom.*

¶*Department of Chemistry, University of Hull, Hull HU6 7RX, United Kingdom.*

§*Current address: Université de Lyon, École Normale Supérieure de Lyon, CNRS, Laboratoire de Physique, F-69342 Lyon, France.*

E-mail: v.garbin@tudelft.nl

Crystal shape and size in the oleofoam

A sample of oleofoam was observed using microscope objective lenses of magnification up to 100×. Typical micrographs are shown in Figure S1(a-b), which suggest that the bulk oleogel network to consist of micrometric needle-like crystals in the oil matrix, up to $\sim 10\ \mu$m in length. To examine the interfacial layer, bubbles were isolated from the oleofoam and re-suspended in oil to remove the bulk crystal network. The sample was pressed between two glass plates until the bubble was flattened to a “pancake” shape. The bubble is shown in Figure S1(c-d). The interface is composed of crystal particles of different shapes, with typical dimensions in the range $1 - 10\ \mu$m. This interfacial layer is formed during rapid cooling leading to heterogeneous nucleation, with simultaneous addition of crystals coming from the bulk during turbulent mixing.

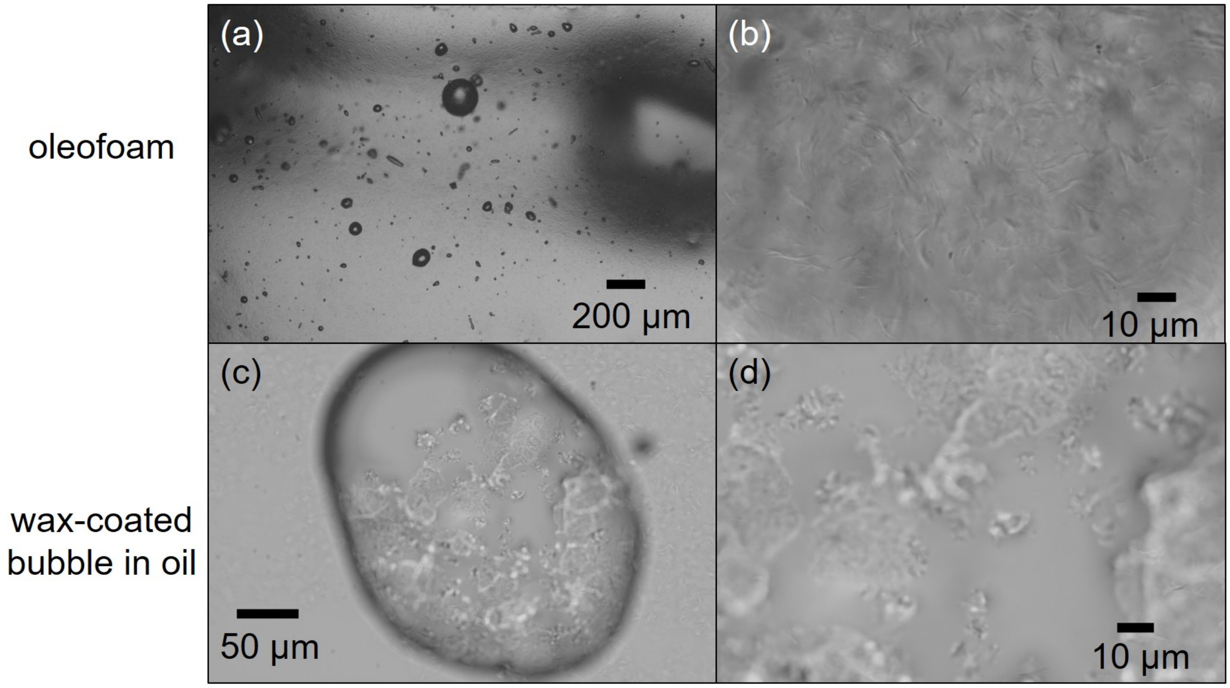

Figure S1: **Micrographs of crystal shapes and sizes.** (a) Oleofoam sample as made following the protocol in Methods. (b) Observation of bulk crystals using 100× magnification. (c) A bubble extracted from the oleofoam, and re-suspended in oil to remove bulk crystals. The bubble is pressed between two glass plates to visualise the interface with high magnification. (d) A high-magnification view of the same bubble interface.
